# The effect of surgical complexity on hearing preservation during cochlear implantation with sheathed perimodiolar electrodes

**DOI:** 10.1007/s00405-025-09581-9

**Published:** 2025-08-07

**Authors:** Ralf Greisiger, Stephen O’Leary, Christofer Bester, Hilde Korslund, Muneera Iftikhar, Greg Eigner Jablonski

**Affiliations:** 1 Department of Otorhinolaryngology and Head & Neck Surgery, Oslo University Hospital, Sognsvannsveien 20, Oslo, NO-0464 Norway; 2 Intervention Centre, Oslo University Hospital, Oslo, Norway; 3 Institute of Clinical Medicine, University of Oslo, Oslo, Norway; 4 Department of Surgery—Otolaryngology, University of Melbourne, Melbourne, Australia

**Keywords:** Cochlear implants, CT-scan, Electrocochleography, Fluoroscopy, Monitoring, Preserve residual hearing, Trauma

## Abstract

**Purpose:**

The causes of residual hearing loss during cochlear implantation are generally poorly understood. This study sought to understand whether the complexity of this surgical approach related to a loss of residual hearing.

**Method:**

Thirty-four adults underwent implantation with a sheathed perimodiolar electrode, via an extended round window approach. During implantation, there was simultaneous video fluoroscopy, electrocochleography (ECochG) and video from the operating microscope. Three investigators reviewed the data simultaneously. Surgery was classified as straightforward or complex, defined as difficulty with either introduction of the electrode/sheath, advancement of the electrode, or withdrawal of the sheath. ECochG signal amplitude was correlated to intracochlear electrode movements, as determined by video fluoroscopy. The primary outcome was relative hearing loss, at least six weeks after surgery. The impact of surgical complexity on relative hearing loss was assessed. A secondary outcome was the impact of surgical complexity or electrode movement on ECochG signal amplitude.

**Results:**

Complex surgery was associated with significantly worse relative hearing preservation (*p*=0.011), as well as lower maximum ECochG amplitudes (Kruskal-Wallis, chi2 = 4.35, *p*= 0.037). After the electrode had been advanced out of the sheath, sudden fluctuations in ECochG amplitude were related to sudden changes in insertion depth. In non-complex surgeries, the residual hearing was independent of such fluctuations (Kruskal-Wallis, chi2=1.12,*p*=0.289).

**Conclusion:**

These data suggest residual hearing is lost early during the implant procedure, following complex surgical events. The low ECochG amplitudes encountered in complex surgeries support this interpretation. ECochG amplitude fluctuations were not associated with poorer residual hearing.

## Introduction

Residual hearing preservation is a goal of cochlear implantation to preserve cochlear function and structure, facilitate electroacoustic hearing, and enrich environmental or music awareness. One critical element of hearing-preserving surgery is the electrode design. Lateral-wall electrodes are thin and flexible and can usually be inserted via the round window to negotiate the complex anatomy of the crista fenestra [1]. Still, their main drawback for hearing preservation is that they inevitably contact the basilar membrane if inserted deeply enough into the cochlea [2]. This limitation is overcome by the perimodiolar electrode, which avoids basilar membrane contact, even when inserted deeply into the cochlea, by traversing the medial cochlear wall [3]. It has been suggested that this design characteristic should make the perimodiolar electrode a better candidate for hearing-preservation, given that deep electrode insertion (and good coverage of the spiral ganglion by electrical stimulation) can be achieved [4], without compromising basilar membrane mobility. However, clinical reports to date have not found a consistent advantage for any type of electrode [5–10]. Some groups report that residual hearing is poorer than that seen with lateral wall electrodes [6, 11], others equivalent acoustic hearing, and only a few have reported an advantage [12–14]. The reason(s) why are not entirely clear. Potential factors include the lower flexibility of perimodiolar electrodes, necessitating more complex engineering and delivery systems for deployment, and a greater risk of cochlear injury when their intracochlear trajectory is not ideal. Furthermore, many surgeons prefer to insert these electrodes via a cochleostomy or extended round window approach [15] to negotiate the crista fenestra [1], and this, too, may cause cochlear trauma [16, 17].

This study was motivated by a desire to help identify aspects of implant surgery, and/or electrode design or deployment, that need to be improved for perimodiolar electrodes to achieve better preservation of cochlear function and structure. This paper examines the time(s) during insertion of a sheathed perimodiolar electrode that cochlear function is disturbed, and the intracochlear behaviour of the electrode at those times. This was achieved by simultaneous video recording of the surgical procedure, intracochlear electrocochleography and video fluoroscopy, in patients with pre-operative audiometric thresholds **< 70** dB at 500 Hz. Sudden fluctuations in the amplitude of the ECochG response were viewed as an indication that the cochlear function may have changed, and these fluctuations were related to co-temporal surgical events or electrode movements, with the latter inferred from the video fluoroscopy. Furthermore, the maximum amplitude of the ECochG amplitude observed during insertion was viewed as a global indication of cochlear function during the implant procedure. Absent or exceptionally small ECochG responses throughout the procedure suggest that the cochlear function may have been compromised prior to the commencement of ECochG recording, i.e. before the electrode was advanced from its sheath within the cochlea. We based this premise on prior reports that ECochG threshold tends to be as good as, or better, than audiometric thresholds [18]. Therefore, larger responses are expected with lower thresholds, provided that cochlear mechanics have not been disturbed. Surgery was defined (below) as complex or straightforward. These intraoperative characteristics were related to the hearing preservation, as assessed from post-operative audiometry > 6 weeks after implantation.

## Methods

The general methodologies have been described previously [19] and are summarised in brief below. These methodologies were derived from initial observations on the insertion of the same perimodiolar electrode studied here. The classification of surgical phases used here arose from this previous study. Images of the experimental setup and fluoroscopy are provided in the original article (Figs. 2 and 6 [19]).

Surgical videos were obtained from the operating microscope, from cochlear opening through to coiling of the electrode leads in the mastoid. Video fluoroscopy (two frames per second) was performed with a Siemens Pheno™ Flat-Detector Cone-Beam Computed Tomogram (FD-CB-CT) at all times that the electrode was moving within the cochlea, and during sealing of the round window and coiling of the electrode leads within the mastoid.

Cochlear implantation was undertaken via a mastoidectomy and posterior tympanotomy using the Cochlear CI632 implant, via an extended round window approach, which involved drilling the anteroinferior margin of the round window lip with a small diameter (≤ 1 mm) diameter diamond bur. Implantation of this sheathed perimodiolar electrode was broken down into distinct phases, specifically (1) opening of the cochlea (i.e. the extended round window approach); (2) introduction of the electrode, within its sheath, into the basal turn of the cochlea; (3) advancement of the electrode out of the sheath; (4) withdrawal of the sheath from the cochlea; (5) sealing of the round window with fascia; (6) coiling of the electrode leads within the mastoid.

Electrocochleography (ECochG) was derived from Cochlear Ltd’s implementation of University of Melbourne electrocochleography system (Cochlear Research Platform v 1.0). The video feed from the computer running the ECochG software was captured, to create a video recording. Electrocochleography was performed from the moment that the most apical electrode contacted the perilymphatic fluids (i.e. advancement of the electrode out of its sheath), through to coiling of the electrode leads within the mastoid. For real-time recording, ECochG was recorded from the most apical electrode contact (electrode 22), in response to a 500 Hz duration pure tone burst presented repeatedly at 108 dB. The response presented was a running average of 4 s. The signal was processed such that the responses to stimuli presented at alternating phases (rarefaction and condensation) were averaged, creating the “difference” or so-called DIF response. This maximises the frequency following hair cell response, known as the cochlear microphonic. For analysis of the cochlear microphonic, the signal was bandpass filtered, centred on the fundamental frequency of the 500 Hz stimulus (10% above and below F0, using a 50th order digital bandpass filter). The CM amplitude was then derived using a fast Fourier transform that was zero-padded to 1,000 samples.

All three video-streams were synchronised and integrated into a single video in OBS studio (version 28). Building upon our published methodology with this approach [19], each video was analysed by three investigators simultaneously, one with expertise in surgery, another electrocochleography and the other fluoroscopy. This allowed us to identify the interrelationships between the surgery, cochlear function and electrode behaviour. For analysis, the procedure was broken down into the phases described in the previous paragraph. The surgeries were then given a global classification of either straightforward, or complex. Surgery was defined as complex if multiple attempts were required to complete the extended Round Window Membrane (RWM) approach, or there were repeated attempts at either insertion of the sheath, advancement of the electrode out of the sheath, or withdrawal of the sheath. Other surgical events noted for detailed cross-modality review were uncontrolled of sudden movements of the electrode or bumping of the electrode with instrumentation. ECochG amplitude was recorded real-time during insertion. The timing of drops (of > 30%), or sudden changes in amplitude were identified, and the maximum amplitude of the ECochG response observed during insertion was noted. Electrode movements on video fluoroscopy prompting detailed cross-modality review included those that were sudden or uncontrolled.

Analysis involved relating electrode movement or surgical events, including surgical complexity to indicators of cochlear function, namely ECochG during surgery and loss of residual hearing based upon pure tone audiometry. Residual hearing loss was calculated as a relative hearing loss [20], averaged across the low frequencies (250, 500 and 1000 Hz). The audiometer limits for “no response” were set at 90, 110, 120, 125, 120, 120 and 115 dB for 125, 250, 500, 1000, 2000, 4000 and 8000 kHz, respectively. This relative hearing loss is reported below as a percentage of hearing lost, based on the amount of hearing loss it would be possible to measure from the pre-operative threshold and maximum audiometer limit. The absolute loss in decibel was also reported across the low frequency pure tone average.

## Results

Recordings were made from 34 patients/surgeries, where 20 were females and 14 male. The average age was 60.7 years with a range from 22.4 to 85.4 years. Of these, ECochG could be obtained, or reliably interpreted as absent, from 24. Videos were adequate to assess surgical complexity in 28.

Fluoroscopy was successfully recorded from 29 out of 34 patients. Post-operative audiometry was available for all patients

None of the patients had cochlea malformations or anatomical deviations on high-resolution CT scans, and for these participants the cause of the hearing loss was not identified.

### Surgical complexity and cochlear function

Surgery was defined as complex in 11 of 28 cases classified. Surgical complexity usually involved difficulty getting the electrode sheath into the cochlea, necessitating repeated drilling of the extended round window approach (Subjects 1, 6, 13,25,30), or struggling to remove the sheath (cases 20,24,29). In one case (Subject 13), the electrode became struck when trying to advance the electrode off the sheath, necessitating electrode removal and further drilling of the extended round window. In 1 case, the stapes was bumped with a sudden loss of ECochG (Subjects 7). In one case (Subject 13) there was significant bleeding, requiring multiple episodes of suctioning near the insertion point. Clearance of the blood from the middle ear led to an improvement in the ECochG.

**Fig. 1:**
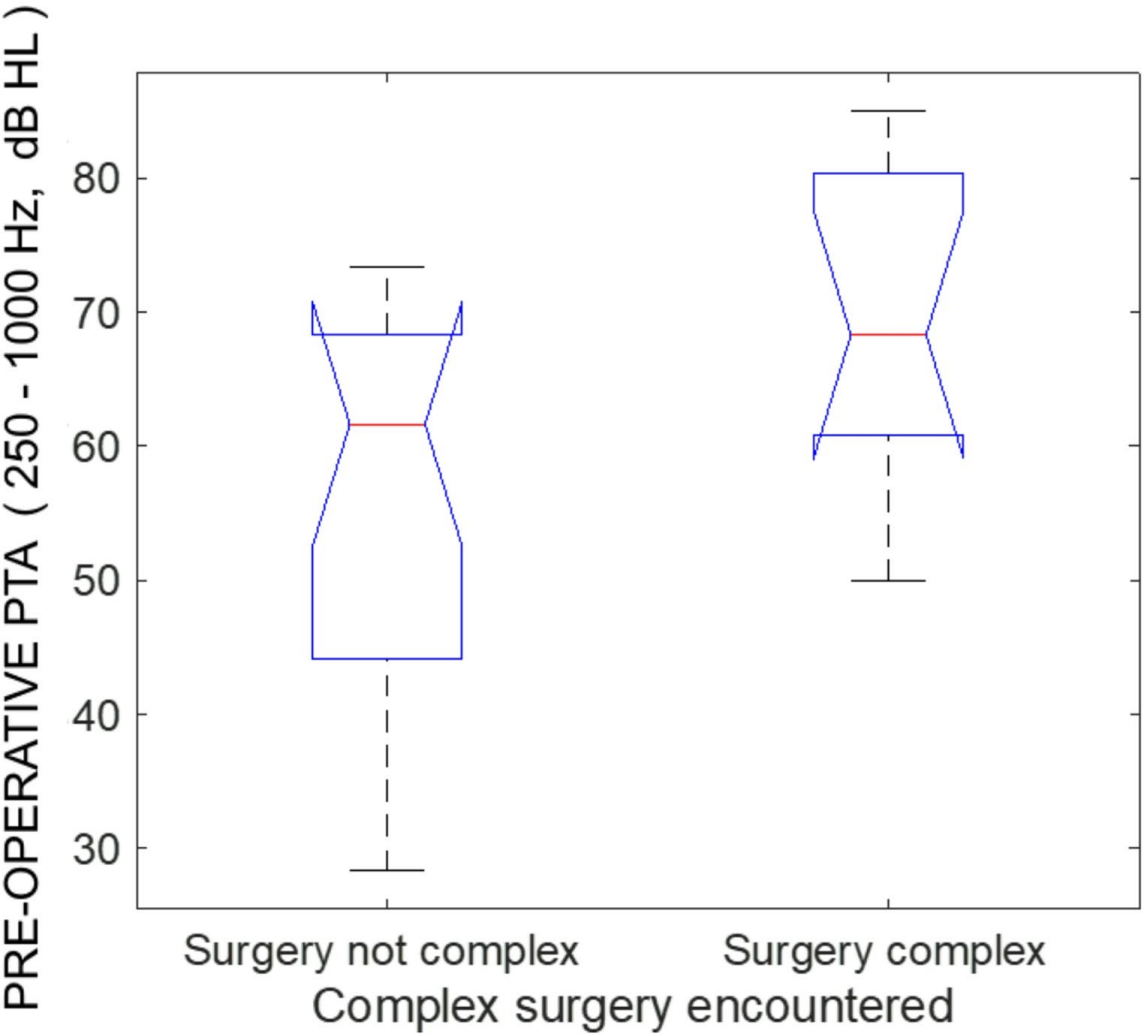
The pre-operative low-frequency pure tone average thresholds, for patients grouped by surgical complexity. The plot presents the median, the interquartile range, and full range, with outliers marked as an “+”

The median pre-operative low-frequency pure-tone average (LF-PTA) was 61.7 dB for non-complex surgery, and 68.3 dB for complex cases. The distribution of LF-PTA was marginally significantly between the groups (Kruskal-Wallis, C^2^ = 4.01, *p* = 0.046; Fig. 1), with hearing skewed towards better hearing for non-complex cases, and poorer hearing for complex cases.

**Fig. 2:**
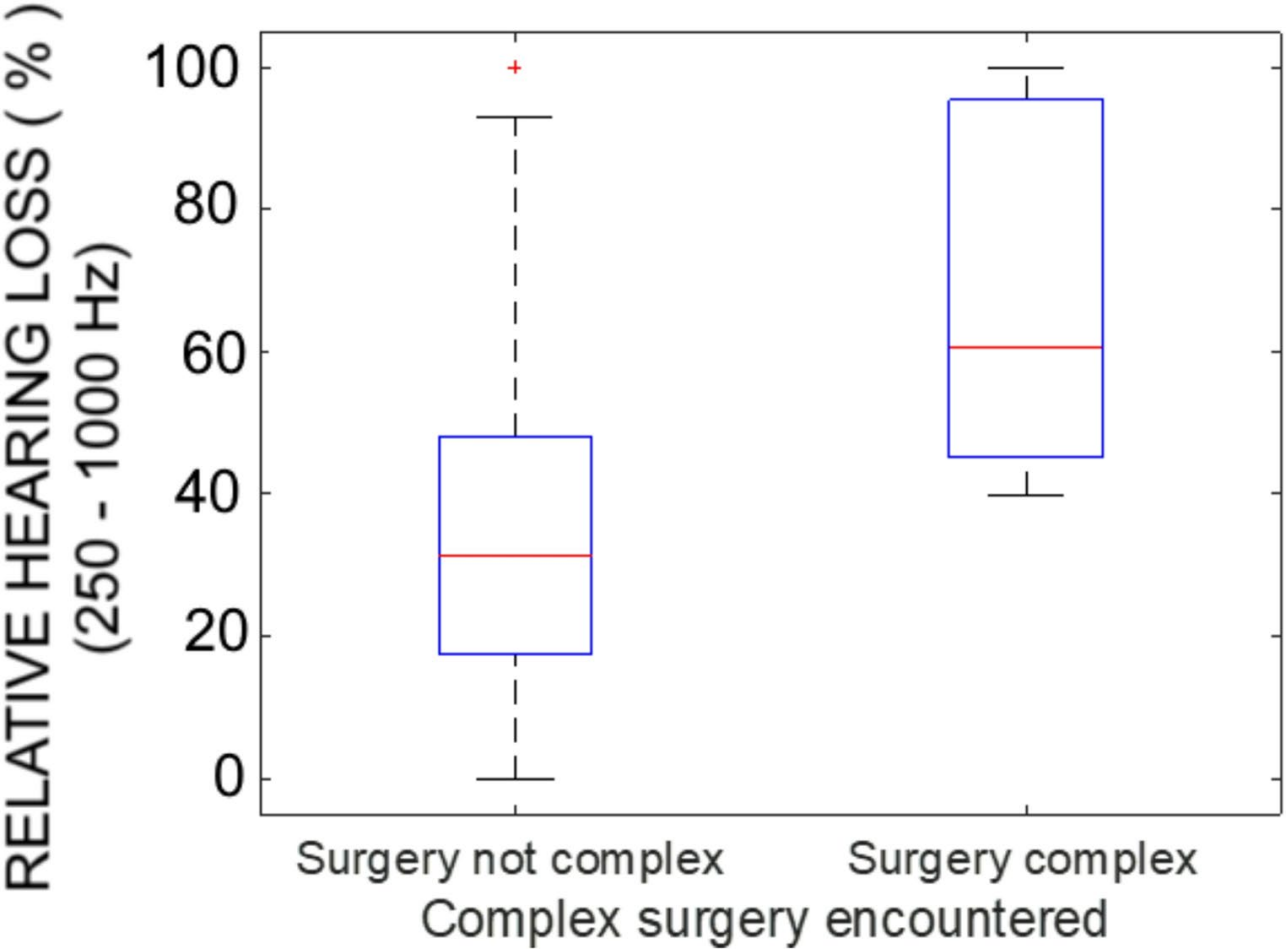
Relative hearing loss over low frequencies (250-1000 Hz) in cases categorised by surgical complexity

The residual hearing was significantly poorer in complex than non-complex surgeries (Fig. 2). The median relative hearing loss was 31% (proportional loss 0.31) for non-complex surgery and 61% for complex surgery, and the distributions were significantly different (Kruskal-Wallis, C^2^ = 7.99, *p* = 0.005).

Because the relative hearing loss calculation may have been influenced by the difference in pre-operative hearing between the groups (with there being greater “potential loss” in the non-complex cases), the hearing loss for each group was plotted in decibels. The low-frequency mean decibel loss for the non-complex surgeries was 21.7 dB, and for complex surgeries 26.7 dB. This difference was not statistically significant (Kruskal-Wallis, C^2^ = 2.13, *p* = 0.15).

**Fig. 3:**
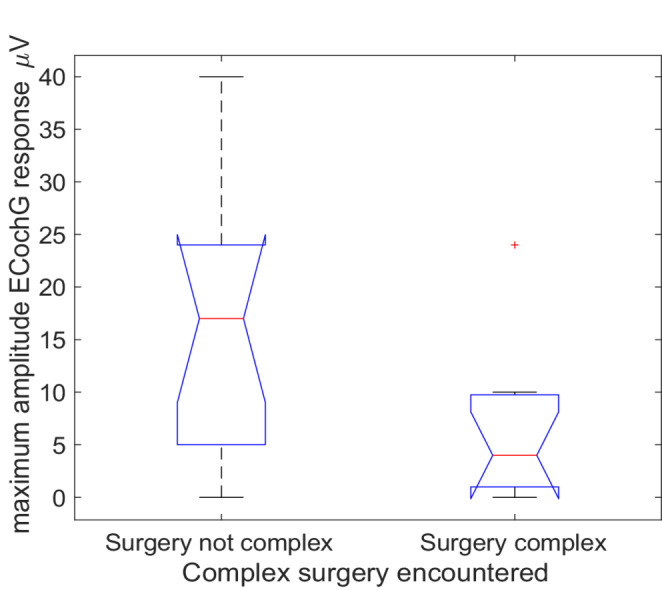
The maximum ECochG amplitude encountered during real-time monitoring during electrode insertion, grouped by the surgical complexity. Boxplot limits as defined in Figure 1

The maximum amplitude of the ECochG response observed during the real-time intraoperative recording was plotted against surgical complexity (Fig. 3). Note that most surgical phases that were deemed to be potentially complex had been completed before the ECochG recording commenced (except for electrode advancement out of the sheath, and sheath withdrawal). The median CM amplitude for non-complex surgery was 17 mV, and for complex surgery 4 mV. In surgically complex cases, the ECochG potential was often too small to be interpreted (< 1 mV), even in cases where the pre-operative thresholds were well below those typically expected to show a strong ECochG, such as the three complex cases with < 1 mV responses with pre-operative low-frequency hearing at, or better than, 75 dB HL. The distributions of CM amplitude were significantly different (Kruskal-Wallis, C^2^ = 4.39, *p* = 0.037).

**Fig. 4:**
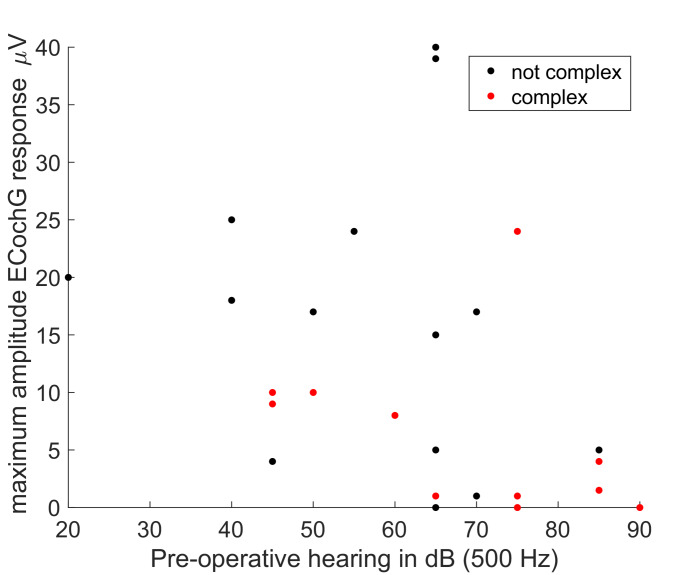
Maximum amplitude of the ECochG response plotted against the pre-operative audiometric threshold at the stimulus frequency of 500 Hz. The points are grouped according to the complexity of the surgery

To determine whether the difference in maximum ECochG amplitudes could be attributed to differences in pre-operative hearing, the pre-operative hearing at 500 Hz was plotted against the ECochG amplitude, with cases grouped by surgical complexity (Fig. 4). As anticipated, ECochG amplitudes were larger when pre-operative thresholds were lower, and while this relationship was present in both groups, it is apparent that maximum ECochG amplitudes were lower in the complex group even within a specific range of relatively low (e.g., 40–60 dB HL) pre-operative thresholds.

### ECochG amplitude fluctuations and electrode movements observed with fluoroscopy

**Fig. 5:**
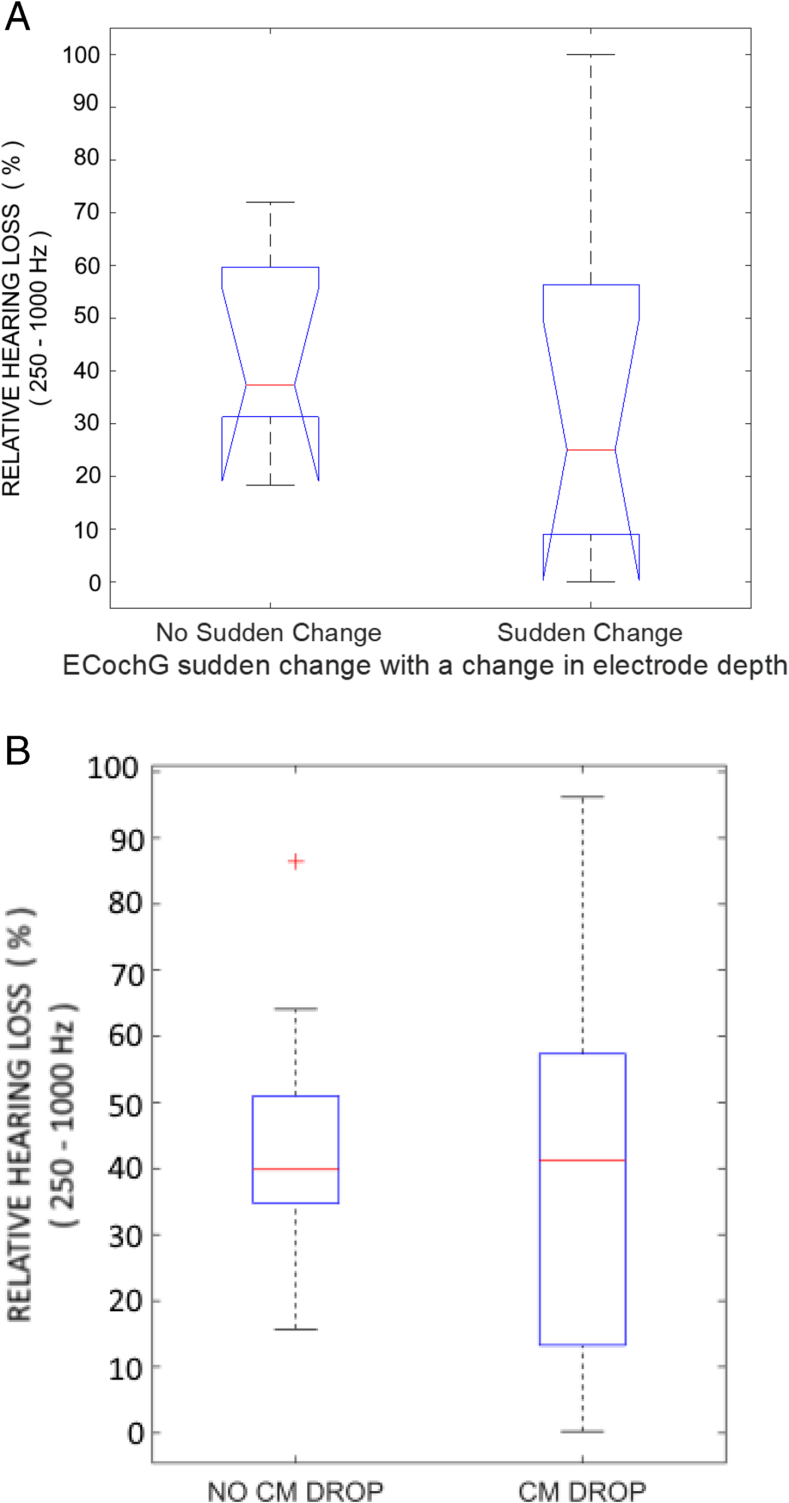
**A**: The relative hearing loss for patients in whom complex surgery was not encountered, classified according to whether they exhibited a sudden change in ECochG amplitude when the electrode suddenly changed depth. **B**: The relative hearing loss for patients with reductions of CM amplitude of at least 30% from a prior maximum at any point during implantation. Red cross for outlier

The general trend was that ECochG amplitudes increased gradually as the electrode was inserted deeper into the cochlea. It is assumed while the tip recording electrode is getting closer to the origin of stimulation (500 Hz) the CM increases. By way of contrast, sudden changes in depth of electrode or its shape were associated with sudden ECochG changes. In most cases, a deeper insertion of the electrode was associated with an in increase in ECochG amplitude and similarly for the converse, shallower insertions (cases 3,5,17, 27), but there were exceptions. In 2 cases (18, 21), a rapid shift deeper by a larger angular depth of insertion (e.g. 60 degrees for case 21), the ECochG decreased in amplitude. In 2 cases (5,17) the electrode bowed outwards towards the outer cochlear wall during sheath removal, and this was associated with a drop in ECochG amplitude. When considering just those patients in whom complex surgery was not encountered, a sudden change in ECochG with a sudden change in electrode depth was associated with similar levels of relative hearing loss to those that did not exhibit this response characteristic (Fig. 5A).

As a possible consequence of these fluctuations with electrode depth, when considering the typical approach of categorizing patients based on the presence of a 30% CM drop at any point during implantation (“CM DROP”) compared with patients with no 30% drop (“NO DROP”), in this group there was no significant difference in hearing preservation between the no drop and CM drop groups (χ2 = 0.05, *p* = 0.82, median relative hearing losses of 40% and 41% respectively) (Fig. 5B).

## Discussion

Surgical complexity was associated with poorer relative hearing loss. On face value, this might suggest that complex surgery is poor for residual hearing, but this interpretation needs to be approached with caution, because patients in whom surgery was complex also had poorer pre-operative residual acoustic hearing. Patients with poorer pre-operative thresholds have less hearing to lose, and this means that their proportional loss for a given decibel drop will be greater. Our observation that decibel drops, while greater in the complex surgery group, did not differ significantly across surgical complexity suggests that this ceiling effect may contribute to the results presented in Fig. 2.

Further evidence that surgical complexity has a negative effect on cochlear function (and ultimately hearing preservation) is found in the CM amplitude results presented in Figs. 3 and 4. When cochlear mechanics are not impeded, and in the absence of auditory neuropathy spectrum disorder, cochlear microphonic (CM) thresholds are very similar to audiometric thresholds [18], and because CM amplitude grows with signal intensity, it is anticipated that maximum CM amplitude encountered during insertion will be larger when pre-operative hearing is better. Figure 4 demonstrates this to be the case. The manifestly smaller ECochG potentials seen in patients with surgical complexity suggests that cochlear function has already been impaired prior to implantation of the cochlear electrode in this group (Fig. 3). That conclusion is supported by Fig. 4, where it is revealed that even when pre-operative thresholds were lower, the CM amplitude was poorer in surgically complex cases.

Based on these observations, we conclude that it is best to avoid surgical complexity as this can adversely impact cochlear function and possibly residual hearing. The most frequently observed causes of surgical complexity (struggling to get the sheath into the cochlea, struggling to remove the sheath, and the electrode buckling out from the sheath during electrode advancement (Subject 13)) were overcome by increasing the size of the extended round window approach. This suggests that the extended round window approach needed to be better defined in the first place. Proper preparation of the extended round window requires sufficient removal of the crista fenestra to establish a straight-line trajectory down the scala tympani of adequate patency (i.e. >0.77 mm – the size of the electrode sheath). Making these judgements is difficult because clinical CT scanning does not provide sufficient anatomical information, leading investigators to explore intraoperative approaches, such as using optical coherence tomography [21] or implementing micro-endoscopes [22, 23] to visualise the local anatomy directly. Successful insertion of the sheath and electrode deployment also requires the adoption of the correct electrode trajectory so that the sheath remains relatively straight as it passes through the hook region. This, too, is a matter of judgement based on the surgeon’s conception of the scalar orientation. Dynamic fluoroscopy can help adjust the sheath’s trajectory during electrode deployment and sheath removal, and this was employed in ~ 25% of cases in this series.

It is unclear why patients with poorer pre-operative hearing tended to have complex surgery. This was most likely a chance occurrence. Another possibility is that knowledge of the pre-operative hearing led to an unconscious bias that affected the surgeon’s technical approach to the operation; they may have been more careful when there was greater residual hearing.

New observations relating to the electrode behaviour when ECochG amplitudes fluctuate were made in this paper. A sudden drop or rise in ECochG amplitude was usually associated with a sudden shift in the angular depth of insertion of the electrode or its shape, as observed in the fluoroscopy. Furthermore, these response characteristics were not associated with poorer residual hearing. These results must be viewed in the context of cochlear mechanics. An increase in amplitude growth of the CM as the cochleotopic place is approached is expected, and when the cochleotopic place is passed, the CM amplitude should drop. The electrode design is another key factor in further interpretation. For this perimodiolar electrode, the array passes around the medial wall of the scala tympani, away from the basilar membrane. This means that the electrode is not expected to impede cochlear mechanics, and this may explain why the ECochG amplitude/electrode depth relationships observed here were not associated with a degradation in residual hearing.

CM amplitude drops have different implications when lateral wall electrodes are used: a drop in CM amplitude is associated with poorer residual hearing [24, 25]. We propose that the difference relates to the electrode design: with lateral wall implants, deeper insertion can impair cochlear mechanics by the electrode contacting the basilar membrane [26], while this is not the case for perimodiolar arrays.

### Implications for cochlear implant research and intraoperative monitoring

1. We present evidence that complex surgery may impair cochlear function and possibly residual hearing. This implies that adequate initial preparation of the surgical access to the cochlea, using soft-surgical principles, is necessary for good preservation of cochlear function. It is encouraging that when this was achieved in our study, hearing preservation was similar to that observed with the insertion of lateral wall electrodes through the round window (~ 70% preservation [19]). This supports the notion that hearing preservation is a matter of the surgical technique, not the perimodiolar electrode design.
2. An absent or small maximum ECochG amplitude observed during the insertion of a sheathed perimodiolar electrode suggests that cochlear function was impaired before the electrode was advanced out of its sheath. We propose that surgeons use this ECochG measure to provide immediate feedback on the quality of their soft surgery and insertion-trajectory estimation.
3. Sudden changes in ECochG amplitude are likely to reflect a sudden change in the depth of insertion of the electrode. In general, it would be advisable to avoid such movements as this would reflect better surgical practice, even though we found no evidence that this affects residual hearing for the perimodiolar electrode.

### Limitations of this study

The technical complexity of this experiment meant that not all data could be collected on each patient, and this reduces the inference possible from these data. A detailed anatomical analysis of the cochlea was not undertaken. If available, this may have provided further insights into why complex surgeries tended to have poorer pre-operative residual hearing.

## Conclusions

Surgical complexity arises when less-than-ideal preparation of the surgical access to the cochlea before the insertion of a perimodiolar electrode. In this situation, the deployment of the electrode may not be smooth, and repeated surgical manipulation, revision of the surgical approach, or attempted insertion may be necessary. This was associated with poorer residual hearing, most likely from a reduction in cochlear function before electrode insertion.

Electrocochleography provides feedback to the surgeon on whether surgical access to the cochlea has impaired cochlear function. When there is a small or absent response, loss of cochlear function is suggested. CM amplitude fluctuations after electrode deployment detect changes in the angular depth of insertion of the apical electrodes; these may not be traumatic but are probably best avoided.

Fluoroscopy then provides an excellent tool for adjusting the electrode trajectory and identifying issues with electrode deployment.

## Data Availability

The data that support the finding of this study are not publicly available due to legal and ethical reasons but are available from the corresponding author RG upon reasonable request.
